# Neuro‐Cancer Interactions Shape Glioma Intratumoral Heterogeneity

**DOI:** 10.1002/advs.202506694

**Published:** 2025-08-21

**Authors:** Tong Wu, Peiran Zhao, Zheng Chen, Tianlei Ying, Zhihong Nie, Wei Hua, Huiyan Li, Ying Mao, Wenbo Bu

**Affiliations:** ^1^ College of Smart Materials and Future Energy State Key Laboratory of Molecular Engineering of Polymers Fudan University Shanghai 200438 P. R. China; ^2^ Department of Neurosurgery Huashan Hospital, Shanghai Medical College Fudan University Shanghai 200040 P. R. China; ^3^ National Center for Neurological Disorders Shanghai 200040 P. R. China; ^4^ Shanghai Key Laboratory of Brain Function and Restoration and Neural Regeneration Shanghai 200040 P. R. China; ^5^ Neurosurgical Institute of Fudan University Shanghai 200040 P. R. China; ^6^ Shanghai Clinical Medical Center of Neurosurgery Shanghai 200040 P. R. China; ^7^ Department of Medical Microbiology, MOE/NHC/CAMS Key Laboratory of Medical Molecular Virology, School of Basic Medical Sciences Fudan University Shanghai 200032 P. R. China; ^8^ The State Key Laboratory of Molecular Engineering of Polymers and Department of Macromolecular Science Fudan University Shanghai 200438 P. R. China; ^9^ Terrence Donnelly Centre for Cellular and Biomolecular Research University of Toronto Toronto M5S 3E1 Canada

**Keywords:** cancer neuroscience, cellular phenotype, functional adaptation, intratumoral heterogeneity, transcriptional states

## Abstract

Glioma intratumoral heterogeneity remains a critical barrier to effective treatment, driving recurrence and resistance to therapy. Emerging evidence suggests that glioma cells acquire neural‐like features through paracrine and synaptic communication with neural cells, fostering functional diversity within tumors. While neuro‐cancer interactions are implicated in glioma heterogeneity, their precise roles remain incompletely synthesized. Here, by consolidating these discoveries, a conceptual framework is proposed for understanding the glioma intratumoral heterogeneity shaped by neuro‐cancer interactions: cellular phenotypes, spanning neurogliomal synapses, glioma networks, and neuronal‐like motility; and transcriptional states, which exhibit remarkable resemblance to neural cells. These diverse cellular phenotypes and transcriptional states synergistically fuel glioma progression, invasion, and resistance. By emphasizing the converging phenotypical and transcriptional evidence with spatial context within them, an underexplored but critical role of neuro‐cancer interactions are proposed in glioma intratumoral heterogeneity. This provides potential strategies to explore and disrupt these neuro‐cancer interactions, offering new insights to address glioma intratumoral heterogeneity for improved therapeutic outcomes.

## Graphical abstract

This review proposes a conceptual framework for understanding glioma intratumoral heterogeneity shaped by neuro‐cancer interactions: functional patterns and transcriptional states. Functional patterns manifest as three major neural‐like adaptations with spatial and potentially hierarchical relationships, including neurogliomal synapses, glioma networks, and neuronal‐like motility. Transcriptional states, resembling their non‐malignant cell counterparts, are associated with these cellular phenotypes and contribute to glioma intratumoral heterogeneity.

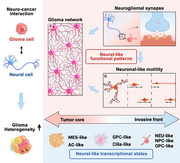

## 1 Introduction

Gliomas, including the highly aggressive glioblastoma (GBM), represent a formidable clinical challenge, with a five‐year survival rate of only 6.9%.^[^ ^1^ ^]^ A major challenge in glioma therapy is intratumoral heterogeneity (ITH), which represents integration of input from genetic,^[^ ^2^, ^3^, ^4^ ^]^ epigenetic,^[^ ^5^, ^6^ ^]^ transcriptional,^[^ ^7^, ^8^, ^9^, ^10^, ^11^ ^]^ and phenotypic heterogeneity.^[^ ^12^ ^]^ This ITH enables clonal selection of resistant subpopulations, fueling recurrence and therapy resistance.^[^ ^13^ ^]^ Despite advances in glioma classification and personalized treatments,^[^ ^14^ ^]^ overcoming this ITH remains a critical obstacle to improving patient outcomes.

Recent evidence has identified normal neural cells as a key driver of ITH.^[^ ^15^, ^16^, ^17^, ^18^ ^]^ Glioma exhibits neural‐like characteristics at both phenotypical^[^ ^19^, ^20^, ^21^ ^]^ and transcriptional^[^ ^7^, ^8^, ^9^, ^11^ ^]^ levels, a phenomenon orchestrated by dynamic neuro‐cancer interactions within the tumor microenvironment.^[^ ^22^, ^23^, ^24^ ^]^ These interactions lead to phenotypic adaptations such as neurogliomal synapses, glioma networks, and neuronal‐like motility (hereafter referred to as functional patterns), as well as transcriptional reprogramming of glioma cells.^[^ ^15^, ^16^, ^25^, ^26^ ^]^ Importantly, these adaptations enhance glioma proliferation, infiltration, and therapeutic resistance, posing significant challenges to current treatment.

**Figure 1:**
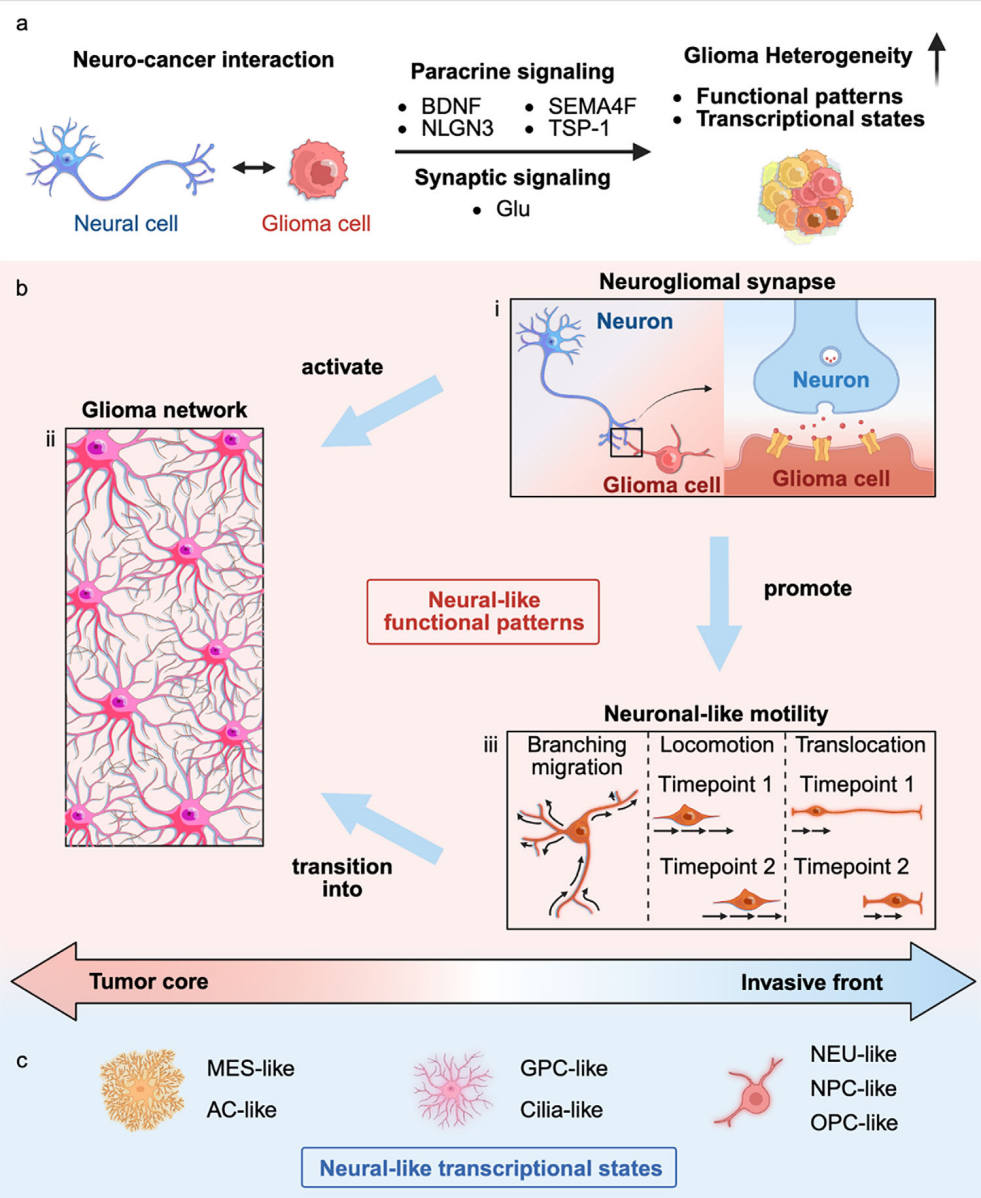
Neuro‐cancer interactions promote glioma intratumoral heterogeneity. a). Glioma intratumoral heterogeneity is shaped by both paracrine and synaptic crosstalk between neural cells and glioma cells. Brain‐derived neurotrophic factor (BDNF) activates tropomyosin‐related kinase B (TrkB) signaling and enhances α‐amino‐3hydroxy‐5‐methyl‐4‐isoxazole propionic acid receptor (AMPAR)‐mediated synaptic strength and connectivity.^[^ ^16^ ^]^ Neuroligin‐3 (NLGN3), released upon cleavage by a disintegrin and metalloproteinase domain‐containing protein 10 (ADAM10), induces malignant synaptic gene expression.^[^ ^17^ ^]^ Remote neuronal activity communicates with glioma cells through semaphorin‐4F (SEMA4F), leading to the emergence of multiple malignant subpopulations characterized by elevated synaptic gene expression.^[^ ^15^ ^]^ Thrombospondin‐1 (TSP‐1) secreted by glioma cells further promotes the formation of neurogliomal synapses.^[^ ^28^ ^]^ Finally, glutamate (Glu)‐mediated neurogliomal synaptic signaling induces calcium transients in glioma cells, driving neuronal‐like motility.^[^ ^29^ ^]^ b). Neural‐like functional patterns of glioma cells: i). Neurogliomal synapse: Synaptic connections between glioma cells and neurons facilitate neuron‐to‐glioma signaling, reinforcing neural‐like functional patterns. ii). Glioma network: Glioma cells form interconnected cellular networks that mimic astrocytic and neuronal structures, enhancing multicellular communication and promoting tumor progression. iii). Neuronal‐like motility: Glioma cells migrate through distinct patterns—branching migration, locomotion, and translocation—demonstrating adaptability in neural environments. Spatially, neurogliomal synapses and glioma cells exhibiting neuronal‐like motility are frequently detected at invasive fronts,^[^ ^19^, ^29^ ^]^ whereas glioma networks compose tumor cores.^[^ ^20^, ^29^, ^30^ ^]^ Neurogliomal synaptic signaling activates glioma networks and promotes neuronal‐like motility,^[^ ^19^, ^26^, ^29^, ^31^ ^]^ and migratory glioma cells with neuronal‐like motility gradually transition into glioma networks.^[^ ^29^ ^]^ c). Neural‐like transcriptional states of glioma cells: mesenchymal (MES)‐like and astrocyte (AC)‐like cell states are highly connected in tumor cores. In contrast, neuronal (NEU)‐like, neural‐progenitor‐cell (NPC)‐like, and oligodendrocyte‐progenitor‐cell (OPC)‐like cells are often invasive and sparsely connected. Glial‐progenitor‐cell (GPC)‐like and cilia‐like cells show intermediate connectivity.^[^ ^25^ ^]^

Significant advances in cancer neuroscience have uncovered dynamic neuro‐cancer interactions.^[^ ^23^, ^24^ ^]^ Meanwhile, single‐cell transcriptomics enables extensive profiling of clinical glioma ITH.^[^ ^8^, ^9^, ^11^, ^25^, ^27^ ^]^ However, an integrative understanding of how neural activity shapes glioma ITH remains lacking. This review addresses this gap by integrating the accumulating evidence into both phenotypical and transcriptional levels: neural‐like functional patterns and cell states (**Figure** **1**a). We focus on three representative functional patterns regulated by neural signaling– neurogliomal synapses, glioma networks, and neuronal‐like motility–with spatial and potentially hierarchical relationships among them. These adaptations contribute to glioma proliferation, invasion, and therapy resistance. We further discuss emerging evidence that transcriptional cell states are shaped by interactions with non‐malignant cells in the tumor microenvironment.^[^ ^25^ ^]^ Together, our review offers a novel conceptual framework for understanding how neuro‐cancer interactions drive glioma intratumoral heterogeneity. Notably, how these phenotypic and transcriptional heterogeneity, along with other underexplored levels of heterogeneity, collectively result in glioma ITH requires further experimental evidence. We hope this framework can serve to deepen our understanding of glioma ITH and propose potential therapeutic strategies to disrupt neuro‐cancer crosstalk and improve clinical outcomes.

## 2 Neuro‐Cancer Interactions Shape Glioma Functional Pattern Heterogeneity

### 2.1 Neurogliomal Synapse

Synapses were traditionally considered exclusive to neuronal communication, primarily occurring between neurons and neuron‐to‐oligodendrocyte precursor cells.^[^ ^23^, ^32^ ^]^ However, it has recently been found that gliomas are integrated into neural circuits across the brain, engaging in widespread functional communication through neuron‐to‐glioma (neurogliomal) synapses.^[^ ^19^, ^31^, ^33^ ^]^ The formation and strengthening of these neurogliomal synapses are promoted by paracrine signaling from both neural cells^[^ ^16^ ^]^ and tumor cells.^[^ ^28^ ^]^ Activity‐dependent secretion of brain‐derived neurotrophic factor (BDNF) from neural cells binds to tropomyosin‐related kinase B (TrkB) on glioma cells, activating CaM kinase II (CAMKII) calcium signaling, facilitating the phosphorylation and subsequent trafficking of α‐amino‐3hydroxy‐5‐methyl‐4‐isoxazole propionic acid (AMPA) receptors (AMPARs) to the postsynaptic membrane, thereby strengthening the synapses. BDNF signaling also increases malignant synaptic connectivity, though the mechanisms require further investigation.^[^ ^16^ ^]^ Moreover, other activity‐regulated neural paracrine signaling molecules, such as neuroligin‐3 (NLGN3)^[^ ^17^ ^]^ and semaphorin‐4F (SEMA4F),^[^ ^15^ ^]^ can also promote synaptic gene expression. On the other side, malignant paracrine factor thrombospondin‐1 (TSP‐1) plays a key role in neurogliomal synapse formation as well.^[^ ^28^ ^]^ TSP‐1 is known as a synaptogenic factor secreted by astrocytes in physiological conditions.^[^ ^34^ ^]^ However, a glioma subpopulation with a distinct gene expression profile that is associated with neural circuit assembly aberrantly secretes TSP‐1, promoting both neuron‐to‐glioma and neuron‐to‐neuron synaptic connectivity.^[^ ^28^ ^]^ Consistently, this subpopulation is enriched in regions of high functional connectivity with the normal brain, highlighting a functional pattern‐associated localizational heterogeneity in gliomas.^[^ ^28^ ^]^

There are diversified subtypes of neurogliomal synapses within a single tumor: the existence of AMPAergic,^[^ ^19^, ^31^ ^]^ gamma‐aminobutyric acid (GABA)‐ergic,^[^ ^35^ ^]^ and cholinergic^[^ ^26^, ^33^ ^]^ synapses has been validated both morphologically and electrophysiologically; glioma cells exhibit electrical responsiveness to adenosine triphosphate (ATP) and dopamine;^[^ ^33^ ^]^ and patient‐derived glioma cells express genes related to nicotinergic, muscarinergic, and adrenergic synapses.^[^ ^33^ ^]^ These synapse subtypes promote glioma progression with various mechanisms: AMPAergic synapses induce calcium transients;^[^ ^19^ ^]^ the combination of GABA to GABA_A_ receptor opens sodium‐potassium‐chloride cotransporter 1 (NKCC1),^[^ ^35^ ^]^ and acetylcholine binds to metabotropic cholinergic receptor muscarinic 3 (CHRM3),^[^ ^26^ ^]^ both increasing intracellular chloride concentration. Moreover, the impact of neurogliomal synaptic signaling is highlighted by the glioma cellular context. While GABAergic neurogliomal synapses are pro‐oncogenic in diffuse midline gliomas (DMGs),^[^ ^35^ ^]^ they are inhibitory for GBM progression.^[^ ^33^ ^]^ Notably, a distinct GABAergic glioma subpopulation in isocitrate dehydrogenase (IDH)‐mutant gliomas has been found capable of firing action potentials and correlates with better survival outcomes.^[^ ^36^ ^]^ How neuro‐cancer interactions differentially shape these heterogeneous neurogliomal synapses remains an open question. In addition to a manifestation of glioma ITH shaped by neuro‐cancer interactions, neurogliomal synapses also mediate neuro‐cancer interactions to further enhance glioma ITH, in turn, such as neuronal‐like motility.^[^ ^21^ ^]^ Therefore, exploring these neuro‐cancer interactions will fuel a deeper understanding of glioma ITH.

### 2.2 Glioma Network

Tumor network is one of the most prominent features of gliomas, resembling networks of astrocytes or immature neurons. The formation of these networks relies on gap junctions^[^ ^20^ ^]^ and adherens junctions^[^ ^37^ ^]^ between tumor microtubes (TMs). Neuronal paracrine signaling promotes TM formation and extension via TSP‐1 in glioma cells,^[^ ^28^ ^]^ orchestrating the known role of TSP‐1 in TM formation.^[^ ^38^ ^]^ The expression of other key genes involved in TM formation, including growth‐associated protein 43 (GAP43),^[^ ^20^ ^]^ connexin 43 (Cx43),^[^ ^20^ ^]^ and tweety‐homolog 1 (TTYH1),^[^ ^39^ ^]^ is also promoted by neural paracrine signaling molecules such as NLGN3^[^ ^17^ ^]^ and BDNF,^[^ ^16^ ^]^ reinforcing glioma network connectivity. Moreover, functional coupling between glioma and astrocyte networks has also been observed,^[^ ^21^ ^]^ though its impact on glioma ITH remains unclear.^[^ ^21^ ^]^

Glioma networks display heterogeneity in their connectivity, function, and dynamic remodeling, driven by neuro‐cancer interactions that shape distinct glioma subpopulations and promote therapy resistance. In gliomas containing TMs, cells in the tumor core form densely interconnected networks that synchronize cellular behavior, enhance metabolic support, and reinforce stress resilience.^[^ ^20^ ^]^ Two morphological variants exist in glioma networks: the filamentous one reaching deeply into the brain parenchyma, and the compact linear one in the perivascular niche.^[^ ^37^ ^]^ Specifically, around 5% of cells in the network exhibit exceptionally high connectivity, a characteristic that is rare in random networks of similar size, indicating scale‐free and small‐world properties of glioma networks.^[^ ^30^ ^]^ The cells in these tumor hubs play a critical role in glioma proliferation.^[^ ^30^ ^]^ In contrast, cells at the invasive front are sparsely connected and more functionally independent.^[^ ^20^ ^]^ This spatial compartmentalization highlights how neuro‐cancer interactions and the tumor microenvironment shape distinct subpopulations that contribute to glioma ITH.

Functionally, glioma networks embody heterogeneity in supporting tumor survival and therapy resistance due to the distinct role of TMs across different tumor compartments. TMs can propagate calcium waves and transport mitochondria and microvesicles, hence providing metabolic support and buffering cytotoxic damage through multicellular communication.^[^ ^20^, ^30^, ^31^ ^]^ These functions are more prominent in the tumor core, where highly interconnected cells exhibit significant resilience to radiotherapy.^[^ ^20^ ^]^ In contrast, the invasive front shows reduced network support. Although this more independent cellular organization is better suited for rapid migration, these cells are more susceptible to damage. This functional disparity illustrates how connectivity heterogeneity reinforces specialization, creating resilience in core cells while maintaining independent invasive cells that may evade treatment and drive recurrence. Moreover, additional distinct functions, such as the capability of rapid repair upon regional ablation^[^ ^20^ ^]^ and the emergence of autonomous rhythmic activity,^[^ ^30^ ^]^ are also highly correlated with the dense interconnectivity of glioma cells. Such spontaneous periodic calcium transients are synchronized throughout the tumor network, benefiting from high connectivity. This synchronization promotes integrated proliferation and survival,^[^ ^30^ ^]^ further complicating clinical interventions.

The heterogeneity of glioma networks creates distinct functional compartments that support therapy resistance and tumor resilience. For example, periodic glioma cells at the most interconnected tumor hubs synchronize the autonomous calcium transients throughout the network to promote glioma proliferation, the highly connected glioma cells in the tumor core coordinate stress responses, while sparsely connected cells at the invasive edge evade treatment and contribute to infiltration.^[^ ^20^ ^]^ Disrupting gap junction proteins may weaken glioma networks, improving therapeutic outcomes.^[^ ^30^ ^]^ By targeting these interconnected structures, therapies may overcome the spatial and functional heterogeneity that drives glioma progression, particularly in recurrent tumors.^[^ ^20^ ^]^

### 2.3 Neuronal‐Like Motility

In contrast to the previously considered collective invasion,^[^ ^37^ ^]^ the latest study demonstrated that unconnected single glioma cells with unique neuronal‐like motility are the major contributors of brain infiltration.^[^ ^21^ ^]^ This neuronal‐like motility manifests three primary patterns resembling neural precursor cell migration during development:^[^ ^21^, ^40^, ^41^, ^42^, ^43^ ^]^ branching migration, locomotion, and translocation.^[^ ^21^ ^]^ Branching migration involves extensive TM branching, protrusion, and retraction, whereas locomotion and translocation are primarily observed in uni‐ and bipolar GBM cells.^[^ ^21^ ^]^ In locomotion, the soma moves along with the protruding TM, while in translocation, the soma follows after the TM protrusion.^[^ ^21^ ^]^

Neuro‐cancer interactions shape these distinct invasive glioma subpopulations and neuronal‐like motility through synaptic and paracrine signaling.^[^ ^15^, ^16^, ^21^, ^44^ ^]^ AMPAR‐mediated glutamatergic synapses are key drivers of this migratory process.^[^ ^15^, ^16^, ^21^ ^]^ Activity‐dependent AMPAR signaling induces calcium transients in glioma cells, activating cyclic adenosine monophosphate (cAMP) response element‐binding protein (CREB) phosphorylation to promote TM dynamics, migration speed, and glioma invasion.^[^ ^21^ ^]^ Moreover, cholinergic neurogliomal synaptic signaling also induces transcriptional responses including fast‐response genes related to axon guidance and motility, as well as long‐lasting response genes enriched in contractility and migration.^[^ ^26^ ^]^ Along with synaptic communications, paracrine signaling from both local and distal neurons further enhances synaptic activity and neuronal‐like migration, as suggested by the elevated expression of synaptic and axon guidance‐related genes.^[^ ^15^, ^16^ ^]^ It is interesting to learn whether other neural signaling molecules, such as pleiotrophin (PTN), which drives clinical invasion hotspots,^[^ ^44^ ^]^ also contribute to the neuronal‐like migration adaptation.

In summary, neuro‐cancer interactions shape heterogeneous yet widespread functional patterns, which can be generally categorized into a triad: neurogliomal synapses, glioma networks, and neuronal‐like motility. **Table** **1** summarizes the distribution of neural‐like functional patterns across different glioma subtypes. Spatially, glioma networks are typically localized in the tumor core, while neurogliomal synapses and invasive glioma cells exhibiting neuronal‐like motility are predominantly found at the invasive front.^[^ ^19^, ^21^ ^]^ Furthermore, these functional patterns also show a degree of hierarchy. Neurogliomal synapses both result from and amplify neuro‐cancer interactions, representing a key adaptation of gliomas to the neural microenvironment. Synaptic signaling can activate glioma networks by inducing calcium waves in the network,^[^ ^19^ ^]^ promoting tumor growth and potentially resistance.^[^ ^29^ ^]^ Moreover, neurogliomal synapses can also drive the neuronal‐like motility of glioma cells,^[^ ^21^ ^]^ which, progressively, transition into the glioma networks^[^ ^21^ ^]^ (Figure 1b). The spatiotemporal relationships among these function patterns further underscore the complexity of therapy resistance driven by neuro‐cancer interactions. In response to neural signaling, glioma cells not only display diverse malignant behaviors but also exploit neurodevelopmental pathways to enhance their adaptive capabilities, posing significant challenges for clinical interventions.

**Table 1:** Functional pattern distribution in different brain tumors.

Tumor entities	WHO grade^[^ ^14^ ^]^	Neurogliomal synapse	Glioma network	Neuronal‐like motility
GBM	4	✓^[^ ^19^, ^31^ ^]^	✓^[^ ^20^, ^21^ ^]^	✓^[^ ^21^ ^]^
DMG	4	✓^[^ ^31^ ^]^	✓^[^ ^31^ ^]^	Not reported
Astrocytoma	2–4	✓^[^ ^19^ ^]^	✓^[^ ^20^ ^]^	Not reported
Oligodendroglioma	2–3	×^[^ ^19^ ^]^	×^[^ ^20^ ^]^	Not reported
Meningioma	1–3	×^[^ ^19^ ^]^	Not reported	Not reported
Brain Metastases	/	✓^[^ ^45^ ^]^ (Tripartite synapse)	✓^[^ ^46^ ^]^ (Cx43 reported)	Not reported

Abbreviations: GBM: glioblastoma; DMG: diffuse midline glioma; Cx43: connexin 43.

## 3 Neuro‐Cancer Interactions Shape Glioma Transcriptional Cell State Heterogeneity

Neuro‐cancer interactions drive the formation of novel glioma transcriptional cell states. For example, stimulation of remote neuronal activity has been found to induce the emergence of multiple distinct glioma subpopulations.^[^ ^15^ ^]^ Among these, the most enriched subpopulation exhibited a unique gene expression profile related to Gene Ontology terms such as glutamatergic synapses and axon guidance.^[^ ^15^ ^]^ In addition to experimental models of neuro‐cancer interactions, single‐cell RNA sequencing (scRNA‐seq) of clinical glioma samples similarly implicates neural and glial cells in shaping malignant cell states,^[^ ^25^ ^]^ as described later.

scRNA‐seq studies have systematically classified the transcriptional cell states across malignant glioma subtypes, including GBMs,^[^ ^8^, ^10^, ^25^ ^]^ DMGs,^[^ ^7^, ^47^ ^]^ oligodendrogliomas (IDH‐Os),^[^ ^9^, ^27^ ^]^ and astrocytomas (IDH‐As).^[^ ^27^ ^]^ Similar malignant cell states resembling non‐malignant neural and glial cell types have been identified in gliomas with distinct genetic backgrounds,^[^ ^7^, ^8^, ^9^, ^10^, ^11^, ^48^ ^]^ such as neuronal (NEU)‐like^[^ ^10^ ^]^ and astrocyte (AC)‐like^[^ ^7^, ^8^, ^9^, ^11^, ^48^ ^]^ states. **Table** **2** highlights the diverse but widespread transcriptional cell states identified in various glioma subtypes. Notably, joint analyses with tumor microenvironment (TME) suggest that neural and glial cells, along with other non‐malignant cell types, may serve as determinants of malignant cell states.^[^ ^25^ ^]^ The abundance of malignant and non‐malignant cells is highly correlated, such as NEU‐like glioma cells with neurons, oligodendrocyte‐progenitor‐cell (OPC)‐like glioma cells with OPCs, and glial‐progenitor‐cell (GPC)‐like glioma cells with microglia.^[^ ^25^ ^]^ Ligand–receptor interaction analysis between these malignant and normal cell types further suggested the interaction mechanisms.^[^ ^25^ ^]^ For example, neurons and NEU‐like gliomas were inferred to interact by NLGN–neurexin (NRXN) interaction,^[^ ^25^ ^]^ which is consistent with a reported neuro‐cancer interaction mechanism.^[^ ^17^, ^49^ ^]^ Overall, experimental and computational evidence concordantly support that neuro‐cancer interactions shape the heterogeneous glioma transcriptional cell states.

**Table 2:** Cell state distribution across glioma subtypes.

Tumor entities	Cell states	Proportion ^a)^, ^b)^ ^)^	Cycling cell ratio ^c)^
Glioblastoma^[^ ^25^ ^]^ (adult)	Astrocyte‐like	≈74%	≈0%
	Cilia‐like	≈10%	≈1%
	Glial‐progenitor‐cell‐like	≈33%	≈12%
	Hypoxia	≈40%	≈2%
	Mesenchymal‐like	≈51%	≈5%
	Neuronal‐like	≈26%	≈8%
	Neural‐progenitor‐like	≈22%	≈13%
	Oligodendrocyte‐progenitor‐like	≈64%	≈18%
	Stress	≈23%	≈6%
Diffuse midline glioma^[^ ^7^ ^]^ (adult/paediatric) ^d)^	Cycling	≈12%/≈11%	/
	Oligodendrocyte‐precursor‐like	≈37%/≈45%	≈16%
	Oligodendrocyte‐like	≈5%/≈8%	≈5%
	Astrocyte‐like	≈16%/≈11%	≈3%
	Mesenchymal‐like	≈28%/≈2%	≈14%
Astrocytoma^[^ ^11^ ^]^ (adult)	Astrocyte‐like	≈12%	Not reported
	Oligodendrocyte‐like	≈18%	
	Undifferentiated	≈70%	
	Stem/progenitor		
Oligodendroglioma^[^ ^9^ ^]^ (adult)	Astrocyte‐like	29.74%	0.00%
	Oligodendrocyte‐like	39.25%	0.33%
	Undifferentiated	20.95%	3.87%
	Stem/progenitor	10.07%	4.57%

^a)^ Proportion: For glioblastoma, data represent the overall proportion of cells assigned to specific meta‐programs. Each meta‐program corresponds to a distinct cell state. Some cells correlate with multiple meta‐programs, so that the sum of the proportions is not 100%.^[^ ^25^ ^]^ For other glioma subtypes, the data is the overall proportion of cells in specific cell states.;^[^ ^7^, ^9^, ^11^ ^]^

^b)^ “≈” annotates that the data were extracted from plots using https://automeris.io: the proportions and cycling rates of glioblastoma cell states were obtained from the work of M. Nomura et al. ^[^ ^25^ ^]^, those of diffuse midline glioma cell states from the work of I. Liu et al.^[^ ^7^ ^]^, and the proportions of astrocytoma from the work of A. S. Venteicher et al.^[^ ^11^ ^]^

^c)^ Cycling cell ratio is the ratio of cycling cells in each cell states;

^d)^ The proportion of cell states for diffuse midline glioma (DMG) corresponds to adult or paediatric DMGs, respectively, while the cycling cell ratio for DMG represents the overall ratio without distinguishing between adult and paediatric cases.^[^ ^7^ ^]^

The two layers of heterogeneity shaped by neuro‐cancer interaction–transcriptional cell states and functional patterns–are highly associated. Both experimental and computational analysis reveal that MES‐like and AC‐like states exhibit higher connectivity, while NEU‐, neural‐progenitor‐cell (NPC)‐, and OPC‐like cells had greater motility and invasion scores, with GPC‐ and cilia‐like glioma cell states showing intermediate profiles.^[^ ^21^, ^25^ ^]^ Moreover, NEU‐, NPC‐, and OPC‐like cells are also enriched with signaling pathways such as synaptic activity/transmission and neurotransmitter receptor activity, which strongly indicate their functional involvement in neurogliomal synapses.^[^ ^10^, ^25^ ^]^ In turn, the calcium‐activated potassium channel KCa3.1, a marker gene for periodic glioma cells, is enriched in MES‐like cells,^[^ ^30^ ^]^ and the GABAergic synapse‐related gene expression signature is predominantly exhibited in OPC‐like glioma cells.^[^ ^35^ ^]^ Neuro‐cancer interaction mechanisms that drive functional patterns also involve the upregulation of the marker genes and pathways of these cell states, indicating the transition of cell states. For example, BDNF, NLGN3, and SEMA4F paracrine factors activate synaptic gene expression,^[^ ^15^, ^16^, ^17^, ^49^ ^]^ which is characterized by NEU‐, NPC‐, and OPC‐like cells.^[^ ^25^ ^]^ Despite these associations, the mechanistic link between glioma transcriptional cell states and functional patterns remains an open question.

Glioma cell states not only represent glioma ITH but are also clinically significant. For example, NEU‐like GBMs are associated with poorer survival outcomes,^[^ ^10^ ^]^ and OPC‐ and NPC‐like cells have a higher proportion in GBM patients with epilepsy than those without epilepsy.^[^ ^50^ ^]^ Therefore, understanding how neuro‐cancer interactions shape glioma transcriptional heterogeneity could be key to overcoming the clinical challenges of glioma ITH and improving therapeutic strategies. Notably, while functional patterns bridge the link between neuro‐cancer interactions and glioma transcriptional heterogeneity, direct mechanisms of how neural cells shape glioma cell states remain limited, underscoring the need for further investigation into their biological and clinical significance.

## 4 Discussion and Future Perspectives

Neuro‐cancer interactions shape glioma ITH on both phenotypic and transcriptional levels, driving various functional patterns and cell states. A systems‐level dissection of these mechanisms will reveal key regulatory ITH networks and refine precision therapies.

Despite recent progress, the role of neuro‐cancer interactions in glioma ITH remains poorly understood, with limited studies addressing its underlying mechanisms. Key questions remain: (1) How do neural cells drive glioma subpopulation diversification? (2) Can this diversification be constrained or reversed? (3) How do neural‐dependent subpopulations impact treatment resistance? Addressing these mechanisms will clarify neural‐driven plasticity and identify strategies to disrupt neuro‐cancer crosstalk for lasting clinical benefit.

Future research should prioritize understanding how neural cells drive glioma subpopulation differentiation, shape resistance trajectories, and influence microenvironmental dependencies. Exploring glioma ITH over time and space is crucial to determining how neural signals regulate tumor plasticity, which can be advanced by spatial technologies such as Visium and MERFISH.^[^ ^51^, ^52^, ^53^, ^54^ ^]^ Organoid models that better capture neuro‐immuno‐oncological interactions, as well as recapitulate cell state heterogeneity, will help uncover mechanisms sustaining glioma adaptation.^[^ ^55^, ^56^ ^]^ Understanding how therapeutic resistance emerges under neural influence will be essential for identifying targetable mechanisms. Additionally, strategies to modulate neuro‐oncogenic signaling could provide new opportunities to limit glioma plasticity and counteract adaptive resistance.

The complexity of neural‐related glioma ITH necessitates an interdisciplinary approach.^[^ ^57^ ^]^ Optogenetics provides spatiotemporal control over neural activity, shedding light on bioelectric signals that drive glioma plasticity.^[^ ^58^ ^]^ Synthetic biology enables gene circuit modifications, offering tools to regulate tumor adaptation.^[^ ^59^, ^60^, ^61^ ^]^ However, challenges remain in tumor‐specific activation and immune evasion. Nanotechnology addresses this by using microenvironment‐responsive particles to detect and regulate biochemical and bioelectric signals in real time.^[^ ^62^, ^63^, ^64^, ^65^ ^]^ AI‐driven models synthesize these multimodal datasets to track glioma evolution and optimize therapeutic strategies.^[^ ^66^, ^67^ ^]^ Bridging these disciplines is key to transforming glioma ITH from a treatment barrier to a modifiable target.

Mechanistic insights into glioma heterogeneity shaped by neuro‐cancer interactions are beginning to inform early‐stage translational efforts. **Table** **3** summarizes three major therapeutic directions that reflect this evolving understanding: (1) directly targeting neuro‐cancer interactions that drive glioma ITH, (2) disrupting neural‐like functional patterns such as neurogliomal synapses and glioma networks, and (3) modulating additional pathways proposed in the broader field of cancer neuroscience. Notably, clinical application of these foundational insights remains in its early stages, and no available data currently demonstrate that targeting neuro‐cancer interactions can prevent glioma recurrence or overcome therapeutic resistance. Proof‐of‐concept studies will be essential to assess whether disrupting the crosstalk between neural cells and glioma cells can improve clinical outcomes by mitigating resistance and recurrence.

**Table 3:** Potential therapeutic approaches translate mechanistic insights from cancer neuroscience.

Targets	Mechanistic insights ^a^ ^)^	Potential drugs	Development stage
ADAM10^[^ ^17^ ^]^	(1)	INCB007839^[^ ^68^ ^]^	Phase I^[^ ^68^ ^]^
AMPAR^[^ ^19^, ^29^, ^31^ ^]^	(1), (2)	Perampanel^[^ ^19^ ^]^	Preclinical^[^ ^19^ ^]^
CHRM3^[^ ^26^ ^]^	(1), (2)	Solifenacin^[^ ^69^ ^]^	Potential repurposing
Cx43^[^ ^70^ ^]^	(2)	Meclofenamate^[^ ^71^ ^]^	Phase I/II^[^ ^71^ ^]^
GABA_A_R^[^ ^35^ ^]^	(2), (3)	Gabazine^[^ ^72^, ^73^ ^]^	Potential repurposing
GAP43^[^ ^20^ ^]^	(2)	Not reported	Potential repurposing
IGF1^[^ ^74^ ^]^	(3)	Linsitinib,^[^ ^75^, ^76^ ^]^ cixutumumab^[^ ^77^, ^78^, ^79^ ^]^	Potential repurposing
p110α^[^ ^80^ ^]^	(3)	Alpelisib,^[^ ^81^, ^82^ ^]^ inavolisib^[^ ^83^, ^84^ ^]^	Potential repurposing
PTN^[^ ^44^ ^]^	(1)	Not reported	/
SEMA4F^[^ ^15^ ^]^	(1)	Not reported	/
TrkB^[^ ^16^ ^]^	(1)	Entrectinib^[^ ^85^, ^86^, ^87^ ^]^	Preclinical^[^ ^16^ ^]^
TSP‐1^[^ ^28^ ^]^	(1)	Gabapentin^[^ ^28^ ^]^	Preclinical^[^ ^28^ ^]^

^a)^ (1) represents neuro‐cancer interactions that shape glioma ITH, (2) annotates neural‐like functional patterns, and (3) points to other mechanisms proposed in the field of cancer neuroscience. Abbreviations: ADAM10: a disintegrin and metalloproteinase domain‐containing protein 10; AMPAR: α‐amino‐3hydroxy‐5‐methyl‐4‐isoxazole propionic acid receptor; CHRM3: cholinergic receptor muscarinic 3; Cx43: connexin 43; GABA_A_R: gamma‐aminobutyric acid type A receptor GAP43: growth‐associated protein 43; IGF1: insulin‐like growth factor 1; PTN: pleiotrophin; SEMA4F: semaphorin‐4F; TrkB: tropomyosin‐related kinase B; TSP‐1: Thrombospondin‐1.

The lack of experimental models that can faithfully recapitulate the dynamic neural–glioma ecosystem is partially a obstacle to glioma clinical development. This limitation makes it difficult to evaluate how neural signals influence glioma adaptation and treatment resistance in physiologically relevant contexts. A patient‐derived organoid platform that preserves neuro‐cancer interactions can provide an alternative approach to address this challenge.^[^ ^88^, ^89^ ^]^ This system maintains glioma heterogeneity at phenotypic and transcriptional levels and has shown predictive accuracy in drug response testing across multiple clinical cohorts. They may serve as a valuable tool for the evaluation of therapies informed by cancer neuroscience.

Targeting ITH in glioma offers two key therapeutic strategies: diminishing ITH to improve treatment efficacy and counteract resistance, and targeting evolutionary subpopulations alongside standard therapies for durable tumor control. Glioma evolution involves synaptic remodeling (e.g., BDNF–TrkB axis)^[^ ^16^ ^]^ and bioelectrical reprogramming (e.g., Cx43/KCa3.1 inhibitors),^[^ ^30^, ^70^, ^90^ ^]^ both potential targets for reducing ITH. Entrectinib suppresses the BDNF–TrkB signaling pathway and is expected to block neurogliomal synapses and inhibit the neuronal‐like subpopulations.^[^ ^16^ ^]^ Photoelectric or piezoelectric nanomaterials that generate controllable electric fields at the cell surface under the appropriate light or ultrasound stimulation, can precisely regulate membrane potential^[^ ^91^ ^]^ or directly mediate the internalization of membrane proteins,^[^ ^92^ ^]^ thus altering Ca^2^⁺ oscillations to weaken ITH. Meanwhile, the triarylmethane‐34 (TRAM‐34) inhibitor, by blocking KCa3.1 channels, disrupts Ca^2^⁺ synchronization and glioma network activity, impairing synaptic remodeling and restricting ITH. Since monotherapies drive adaptive resistance, a more effective strategy is to model neurodependency to track ITH dynamics under therapy. For instance, a sequential combination of synaptic inhibitors and immune checkpoint blockade may halt neuro‐driven tumor growth, boost antitumor immunity, and prevent compensatory escape.

A great deal of work remains to be done in understanding glioma ITH and its neural regulation, but this field holds immense potential to transform precision neuro‐oncology and improve patient outcomes. At the same time, studying neural‐driven glioma plasticity may offer broader insights into nervous system regulation of tissue homeostasis, immune modulation, and regeneration, providing a perspective on tumor biology from the lens of neuro‐oncology.

## 5 Conclusion 

Neuro‐cancer interactions are increasingly recognized as central to glioma intratumoral heterogeneity, influencing both cellular phenotypes and transcriptional states. This review provides an integrated framework that connects neural inputs with spatially organized glioma adaptations, highlighting how glioma cells exploit neural cues to drive proliferation, invasion, and resistance.

Future studies should aim to clarify causal links, develop neural‐containing experimental models, and define therapeutic vulnerabilities within this axis. By perceiving glioma as a neural‐adaptive ecosystem, new opportunities may emerge to target plasticity, limit heterogeneity, and ultimately improve clinical outcomes.

## Conflict of Interest

The authors declare no conflict of interest.

## Author Contributions

T.W., P.Z., and Z.C. contributed equally to this work. T.W. performed conceptualization, investigation, visualization, wrote – original draft, and wrote – review and edited the original draft; P.Z. performed funding acquisition, wrote – original draft, and wrote – review and edited the original draft; Z.C. wrote – review and edited the original draft; T.Y.: wrote – review and edited the original draft; Z.N.: wrote – review and edited the original draft; W.H. wrote – review and edited the original draft; H.L. performed conceptualization, funding acquisition, supervision, wrote – original draft, and wrote – review and edited the original draft; Y.M. performed supervision and wrote – review and edited the original draft. W.B. performed supervision and wrote – review and edited the original draft.

## Declaration of Generative AI and AI‐Assisted Technologies in the Writing Process

During the preparation of this work, the author(s) used ChatGPT and Gemini in order to improve the readability and language of the manuscript. After using this tool/service, the author(s) reviewed and edited the content as needed and take(s) full responsibility for the content of the published article.
